# Portmanteau test statistics for seasonal serial correlation in time series models

**DOI:** 10.1186/s40064-016-3167-4

**Published:** 2016-09-05

**Authors:** Esam Mahdi

**Affiliations:** Department of Mathematics, Islamic University of Gaza, Gaza, Palestine

**Keywords:** Diagnostic check, Portmanteau test statistic, Residual autocorrelation function, ARMA models, SARMA models, 62M10, 91B84

## Abstract

The seasonal autoregressive moving average SARMA models have been widely adopted for modeling many time series encountered in economic, hydrology, meteorological, and environmental studies which exhibited strong seasonal behavior with a period *s*. If the model is adequate, the autocorrelations in the errors at the seasonal and the nonseasonal lags will be zero. Despite the popularity uses of the portmanteau tests for the SARMA models, the diagnostic checking at the seasonal lags $1s,2s,3s,\ldots ,ms$, where *m* is the largest lag considered for autocorrelation and *s* is the seasonal period, has not yet received as much attention as it deserves. In this paper, we devise seasonal portmanteau test statistics to test whether the seasonal autocorrelations at multiple lags *s* of time series are different from zero. Simulation studies are performed to assess the performance of the asymptotic distribution results of the proposed statistics in finite samples. Results suggest to use the proposed tests as complementary to those classical tests found in literature. An illustrative application is given to demonstrate the usefulness of this test.

## Background

The multiplicative seasonal autoregressive moving average models, SARMA $(p,q)\times (p_{s},q_{s})_{s}$, for the univariate time series $Z_{t}, t=1,2,\ldots ,n,$ is defined by

1 $$\Phi ({B}^{s})\phi ({B}) Z_{t}= \Theta ({B}^{s})\theta ({B})a_{t},$$

where $\phi ({B}) = 1-\phi _{1} B^{1}-\cdots \phi _p B^{p}$ and $\theta ({B}) = 1-\theta _{1} B^{1}-\cdots \theta _q B^{q}$ are polynomials in *B* of degrees *p* and *q* respectively, whereas $\Phi ({B}^{s}) =1-\Phi _{1} B^{s}-\cdots \phi _{p_{s}} B^{sp_{s}}$ and $\Theta ({B}^{s}) =1-\Theta _{1} B^{s}-\cdots \Theta _{q_{s}} B^{sq_{s}}$ are polynomials in ${B}^{s}$ of degrees $p_{s}$ and $q_{s}$ respectively, *p* and $q\ge 0$ are the order of the non-seasonal autoregressive, AR, model and moving average, MA, model respectively, whereas $p_{s}$ and $q_{s}\ge 0$ are the order of the seasonal autoregressive, SAR, model and seasonal moving average, SMA, model respectively, *B* is the backshift operator on t, and $s>0$ is the length of the seasonal period. The white noise process $a_{t}$ is assumed to be uncorrelated in time with a mean zero; that is, *E*(*a*_*t*_) = **0** and $E(a_{t}a_{t-\ell})=\sigma^{2}\delta _{\ell}$, where $\sigma^{2}$ is the variance and $\delta _{\ell}$ is the usual Kronecker delta with unity at lag $\ell =0$ and zero elsewhere. It is assumed that the model is stationary, invertible and not redundant (Box and Jenkins 1970; Cleveland and Tiao 1976; Brockwell and Davis 2009; Box et al. 2005).

Under the null hypothesis that the model has been correctly identified the residuals, ${\hat{a}}_{t}$, are approximately white noise. When there is no significant autocorrelation in the residuals, their sample autocorrelations, ${\hat{r}_{\ell}=\sum _{t=\ell +1}^{n}\hat{a}_{t} \hat{a}_{t-\ell}}/{\sum _{t=1}^{n}\hat{a}_{t}^{2}}\approx 0,$ for $\ell =1,2,\ldots ,m\le n-1$, where *m* is the largest lag considered for autocorrelation. On the other hand, when there is autocorrelation present, the autocorrelation values should significantly deviate from zero. However, the Box and Pierce (1970) and the Ljung and Box (1978) portmanteau test statistics are commonly used to check the lack of fit of ARMA models (Li 2004); in many situations, they are implemented to check the lack of fit of SARMA models. Using such tests for SARMA models would be misleading and not enough as these tests consider the autocorrelations corresponding to the nonseasonal lags $\le m$ and ignore the possibility of autocorrelations at seasonal lags of multiple period *s*. Despite the popularity of the SARMA models in various economic time series and financial data, the portmanteau tests at seasonal lags $1s,2s,3s,\ldots ,ms\le (n-1)$ where *s* is the seasonal period, has not yet received as much attention as it should deserve. Recently Duchesne (2007), Ursu and Duchesne (2009) considered serial correlation testing in multiplicative seasonal univariate and multivariate time series models. Duchesne (2007) proposed his test statistic based on a kernel-based spectral density estimator of Shin (2004), whose weighting scheme is more adapted to autocorrelations associated to seasonal lags. Complementary statistics for testing whether the seasonal autocorrelations of the series are different from zero are then needed in literature. In particular, for SARMA processes with $p\ll s$ and $q\ll s$ where the roots of the equation $\phi ({B})\theta ({B})=0$ are not close to the unit circle, McLeod (1978) indicated that the residual autocorrelations at the seasonal lags $1s, 2s, \ldots ,ms$, where *m* is any fixed number $\gg 1$, may have the approximately the same covariance matrix as the first *m* residual autocorrelations in the nonseasonal model

2 $$\Phi (B) Z_{t}= \Theta (B)a_{t},$$

where the order of $\Phi (B)$ and $\Theta (B)$ are $p_{s}$ and $q_{s}$ respectively. Motivated by these facts, we introduce a list of new seasonal portmanteau tests that can be used as complementary tests to those classical portmanteau tests found in literature. The proposed tests ignore lags that are not at multiples of the natural period and consider only relevant autocorrelations at multiple period lags $1s, 2s, \ldots ,ms$ so that the seasonal test can gain more power for some cases where data exhibit a very strong seasonal behavior with a period *s* and insignificant correlations at nonseasonal lags.

In the next section, a brief review of commonly univariate portmanteau tests employed for diagnostic checking in ARMA models is given. In "Portmanteau test statistics for SARMA models" section, we modify the usual portmanteau test statistics suggested by Box and Pierce (1970), Ljung and Box (1978), Peña and Rodríguez (2002, 2006), Fisher and Gallagher (2012), Gallagher and Fisher (2015) to the SARMA class. The approximation distributions of the proposed tests are derived in "Asymptotic distributions" section. In "Simulation studies" section provides simulation experiments demonstrating the behaviour of the asymptotic distributions of the proposed test statistics. We close this article with "An empirical application" section by introducing an illustrative application of seasonal data demonstrating the usefulness of the devised tests. We conclude in "Conclusion" section with a discussion.

## Portmanteau test statistics for ARMA models

The diagnostic portmanteau test for the adequacy of fitted ARMA models was introduced by Box and Pierce (1970) based on the asymptotic distribution of the residual autocorrelations, $\hat{r}_{1},\hat{r}_{2},\ldots ,\hat{r}_{m}$, where $m\le n-1$ is the largest selected lag. Their test statistic is

3 $$Q_{m} = n\sum _{\ell =1}^{m}\hat{r}_{\ell}^{2}\sim \chi ^{2}_{m-p-q}.$$

Ljung and Box (1978) improved the finite sample performance of Box and Pierce (1970) by introducing a modified statistic based on standardizing the residual autocorrelations

4 $$\hat{Q}_{m} = n(n+2)\sum _{\ell =1}^{m}(n-\ell )^{-1}\hat{r}_{\ell}^{2}\sim \chi ^{2}_{m-p-q}.$$

Peña and Rodríguez (2002) devised a univariate portmanteau test based on the *m*-th root of the determinant of the Toeplitz residual autocorrelation matrix of order $m+1$,

5 $$\begin{aligned} {\mathcal {\hat{R}}}_{m}=\left( \begin{array}{cccc} 1&{} \hat{r}_{1} &{} \ldots &{} \hat{r}_{m} \\ \hat{r}_{-1} &{} 1 &{} \ldots &{}\hat{r}_{m-1} \\ \vdots &{} \ldots &{} \ldots &{} \vdots \\ \hat{r}_{-m} &{} \hat{r}_{-m+1} &{} \ldots &{} 1 \\ \end{array} \right) , \end{aligned}$$

where $\hat{r}_{-\ell}=\hat{r}_{\ell}$ for all lags $\ell =1,2,\ldots ,m$. They approximated the distribution of their proposed test statistic by the gamma distribution and provided simulation experiments to demonstrate the improvement of their statistic in comparison with the one that is given by Ljung and Box (1978). Peña and Rodríguez (2006) suggested to modify the generalized variance test by taking the log of the $(m+1)$-th root of the determinant of ${\mathcal {\hat{R}}}_{m}$ given in (5). They proposed two approximations by using the Gamma and Normal distributions to the asymptotic distribution of this test and indicated that the performance of both approximations for diagnostic checking in linear models is similar and more powerful for small sample size than the previous one.

Battaglia (1990) noted that the powers of portmanteau tests can be misleading as they falsely decrease as *m* increases. In this light, Lin and McLeod (2006) suggested an improvement to Peña and Rodríguez (2002, 2006) statistics using Monte-Carlo version as they noted that it is quite often that the test statistic does not agree with the suggested Gamma approximation. Mahdi and McLeod (2012) extended Peña and Rodríguez (2002, 2006) and Lin and McLeod (2006) tests to the multivariate time series. Their univariate test statistic is

6 $$\mathfrak {D}_{m}=-3n(2m+1)^{-1}\log |{\mathcal {\hat{R}}}_{m}|\sim \chi ^{2}_{3m(m + 1)(4m + 2)^{-1}-p-q}.$$

Recently, Fisher and Gallagher (2012) provided a portmanteau statistic consisting of a weighted sum of squared of residual autocorrelation terms as follows

7 $$\tilde{Q}_{m}=n(n+2)\sum _{\ell =1}^{m}w_{\ell}(n-\ell )^{-1}\hat{r}_{\ell}^{2},$$

where $w_{\ell}(.)$ are the weights putting more emphasis on the autocorrelations corresponding to the smaller lags. They utilized the approximation similar to Peña and Rodríguez (2002) and derived the limiting distribution of their weighted portmanteau tests as a Gamma distribution. More recently, Gallagher and Fisher (2015) suggested to consider three weighting schemes for the weights in (7). The weighting schemes used in their three statistics were: the squared Daniell kernel-based weights as suggested by Hong (1996a, b), $w_{\ell} =(n+2)(n-\ell )^{-1}K^{2}(\ell /m)$, the geometrically decaying weights, $w_{\ell} =(p+q)a^{\ell -1}$, for some $0<a<1$, and the data-adaptive weights which give the following data-adaptive weights test

8 $$\dot{Q}_{m}=n(n+2)\sum _{\ell =1}^{m_{0}}(n-\ell )^{-1}\hat{r}_{\ell}^{2}+n\sum _{\ell =m_0+1}^{m}w_{\ell}\hat{r}_{\ell}^{2},$$

where the first $m_0$ terms obtain the standardizing weight $(n+2)/(n-\ell )$ from the Ljung-Box statistic, and the remaining weights selected to be summable $w_{\ell} =-\log (1-\mid \hat{\pi}_{\ell} \mid )$, $m_0=\min (\log (n),M)$, where *M* is a finite bound, $\hat{\pi}_{\ell}$ is the residual partial autocorrelation at lag $\ell$ and Daniell kernel function is

$$\begin{aligned} K(u)=\left\{\begin{array}{ll} \sin (\sqrt{3}\pi u)/{\sqrt{3}\pi u}, &{} \quad \hbox {for}\,|u|<1; \\ 0, &{} \quad \hbox {for}\,|u|\ge 1. \end{array} \right. \end{aligned}$$

Gallagher and Fisher (2015) indicated that the weighted portmanteau tests can be more powerful to detect the underfit ARMA models in many situations and less sensitive to the choice of the maximum correlation lag, especially when *m* depends on *n* comparing with the other statistics found from the literature.

## Portmanteau test statistics for SARMA models

Replacing $\hat{r}_{\ell}, \ell =1, 2, \ldots , m$ by $\hat{r}_{\ell s}$, where $\hat{r}_{1 s},\hat{r}_{2 s},\ldots ,\hat{r}_{ms}$ are the residual autocorrelations at the multiple period lags $1s, 2s, \ldots ,ms$, will easily extend the classical portmanteau test statistics to test for seasonality at lags multiple of period *s*. This modification is justifiable under the conditions indicated by McLeod (1978) that we mentioned in the introduction of this article. We devise a list of new portmanteau tests for diagnostic checking of seasonal time series.

The proposed goodness-of-fit tests modify those statistics given in Box and Pierce (1970), Ljung and Box (1978), Fisher and Gallagher (2012) and Mahdi and McLeod (2012) to the SARMA class, respectively, as follows

9 $$Q_{m}(s)= n\sum _{\ell =1}^{m}\hat{r}_{\ell s}^{2}$$

10 $$\hat{Q}_{m}(s)= n(n+2)\sum _{\ell =1}^{m}(n-\ell s)^{-1}\hat{r}_{\ell s}^{2}$$

11 $$\tilde{Q}_{m}(s)= n(n+2)\sum _{\ell =1}^{m}w_{\ell s}(n-\ell s)^{-1}\hat{r}_{\ell s}^{2}$$

12 $$\mathfrak {D}_{m}(s)= -3n(2m+1)^{-1}\log |{\mathcal {\hat{R}}}_{m}(s)|$$

where

13 $$\begin{aligned} {\mathcal {\hat{R}}}_{m}(s) = \left( \begin{array}{ccccc} 1 &\quad {} \hat{r}_{s} &\quad {} \hat{r}_{2 s} &\quad {}\ldots &\quad {} \hat{r}_{ms} \\ \hat{r}_{-s} &\quad {} 1 &\quad {} \hat{r}_{s} &\quad {} \ldots &\quad {} \hat{r}_{(m-1)s} \\ \vdots &\quad {} \ldots &\quad {} \ldots &\quad {} \ldots &\quad {}\vdots \\ \hat{r}_{-ms} &\quad {} \hat{r}_{-(m-1)s} &\quad {} \hat{r}_{-(m-2)s} &\quad {}\ldots &\quad {} 1 \\ \end{array} \right) \end{aligned}$$

It is worth noting that seasonal process has a spectral representation containing a stochastic periodic component with period *s* and non infinitesimal contribution to the variance of the process. Such a periodic component is a linear combination, with random weights, of sines with periods *s* / *j*, where $j = 1/2,\ldots ,s/2$. The corresponding contribution to the autocorrelation is a damped sine wave with period *s*. It follows that the autocorrelation may be affected by seasonality at each lag. Thus, the proposed seasonal tests are expected to provide more power than the classical portmanteau tests found in literature for pure seasonality by ignoring lags that are irrelevant. On the other hand, when the correlations at the nonseasonal lags are presented, the classical nonseasonal tests will outperform the proposed procedure. This restricts the use of the seasonal tests; therefore, we recommend to use the seasonal and nonseasonal test statistics as complementary to each other.

## Asymptotic distributions

The limiting distribution of the resulting seasonal tests are obtained by a straightforward extension of those obtained in Box and Pierce (1970), Ljung and Box (1978), Fisher and Gallagher (2012), Gallagher and Fisher (2015) and Mahdi and McLeod (2012) and are summarized in the following theorems.

### **Theorem 1**

*Assume that the*SARMA $(p,q)\times (p_{s},q_{s})_{s}$*model specified as in* (1) *has i.i.d. innovations*$\{a_{t}\}$*with mean zero and finite constant variance. For constantsmands*, *as*$n\rightarrow \infty$, *where*$ms\le (n-1)$, $p, q \ll s$, *and the roots of the equation*$\phi ({B})\theta ({B})=0$*are not close to the unit circle. When the model has adequately been identified, the test statistics for lack of*SARMA *fit models*, $Q_{m}(s)$*and*$\hat{Q}_(s)$, *would for largenapproximately distributed as*$\chi ^{2}_{m-\nu}$, *where*$\nu =p_{s}+q_{s}$.

### *Proof*

Box and Pierce (1970) showed that the vector of the residual autocorrelations at nonseasonal lags $\sqrt{n}\varvec{\hat{r}_{m}}$ from a correctly identified and fitted ARMA (*p*, *q*) model can be asymptotically distributed as a multivariate normal distribution with mean vector zero and covariance matrix $\varvec{({\mathbb {I}}_{m}-Q)}$, where ${\mathbb {I}}_{m}$ is an identity matrix and $\varvec{Q}$ is a matrix with rank $p+q$. Consider the SARMA model where $p\ll$ and $q \ll s$ and the roots of the equation $\phi ({B})\theta ({B})=0$ are not close to the unit circle. McLeod (1978) indicated that the vector of the residual autocorrelations at seasonal lags $1s,2s,\ldots ,ms$, has approximately the same distribution of the vector of the residual autocorrelations at nonseasonal lags $1,2,\ldots ,m$. Thus, the vector $\sqrt{n}\varvec{\hat{r}_{ms}}$ from a correctly identified and fitted SARMA $(p,q)\times (p_{s},q_{s})_{s}$ model would for large *n* be distributed as a multivariate normal with mean vector zero and covariance matrix $\varvec{({\mathbb {I}}_{m}-Q_{s})}$, where $\varvec{Q_{s}}$ is a matrix with rank $p_{s}+q_{s}$. It follows that both $Q_{m}(s)$ and $\hat{Q}_(s)$ have the same asymptotic distribution as $\chi ^{2}_{m-\nu}$, where $\nu =p_{s}+q_{s}$. $\square$

### **Theorem 2**

*Under the assumptions of * Theorem 1, $\tilde{Q}_{m}(s)$*converges in distribution to*$\sum _{i=1}^{m}\lambda _{i}\chi _{i}^{2}$, *where*$\{\chi _{i}^{2}\}$*denotes a sequence of independent chi-squared random variables, each with one degree of freedom, and*$\lambda _{1},\ldots ,\lambda _{m}$*are the eigenvalues of*$\varvec{({\mathbb {I}}_{m}-Q_{s})M}$*with*${\mathbb {I}}_{m}$*an identity matrix*, $\varvec{Q_{s}}$*is a projection matrix defined as*$\varvec{Q_{s}}=\varvec{X}\Sigma ^{-1}\varvec{X^{\prime}}$, *where*$\Sigma ^{-1}$*is the information matrix for the parameters*$\Phi _{1},\ldots ,\Phi _{ps}$*and*$\Theta _{1},\ldots ,\Theta _{qs}$, *Xis an*$m\times (p_{s}+q_{s})$*matrix defined similar to* McLeod (1978, Eq. (16)) *with elements*$\Phi ^{\prime}$, *and*$\Theta ^{\prime}$*defined by*$1/\Phi (B)=\sum _{i=1}^{\infty}\Phi _{i}^{\prime}B^{i},$*and*$1/\Theta (B)=\sum _{i=1}^{\infty}\Theta _{i}^{\prime}B^{i}$, *and*$\varvec{M}$*is an*$m\times m$*diagonal matrix with diagonal weights*$\{1, (m-1)/m, \ldots ,2/m,1/m\}$.

### *Proof*

The test statistic $\tilde{Q}_{m}(s)$ can be be expressed as quadratic form

$$\tilde{Q}_{m}(s)=n\varvec{\hat{r}_{ms}^{\prime}} \varvec{M} \varvec{\hat{r}_{ms}},$$

where $\varvec{\hat{r}_{ms}}=(r_{s},\ldots ,r_{ms})^{\prime}$ is the $m\times 1$ vector of the autocorrelations at seasonal lags and $\varvec{M}$ is an $m\times m$ diagonal matrix with diagonal elements $\{1, (m-1)/m, \ldots ,2/m,1/m\}$. Using the same argument in the proof of the previous theorem, we notice that the vector $\sqrt{n}\varvec{\hat{r}_{ms}}$ from a correctly identified and fitted SARMA $(p,q)\times (p_{s},q_{s})_{s}$ model would for large *n* be distributed as a multivariate normal with mean vector zero and covariance matrix ${({\mathbb {I}}_{\varvec{m}}-\varvec{Q}_{\varvec{s}})}$, where $\varvec{Q_{s}}$ is a matrix with rank $p_{s}+q_{s}$ and defined as $\varvec{X}\Sigma ^{-1}\varvec{X}^{\prime}$, where *X* is an $m\times (p_{s}+q_{s})$ matrix and $\Sigma _{s}^{-1}$ is the information matrix for the parameters $\Phi _{1},\ldots ,\Phi _{ps}$, and $\Theta _{1},\ldots ,\Theta _{qs}$.

From the theorem on quadratic forms given by Box (1954, Theorem 2.1), the asymptotic distribution of $\tilde{Q}_{m}(s)$, as $n \rightarrow \infty$, is approximated by

14 $$\sum _{i=1}^{m}\lambda _{i}\chi _{i}^{2},$$

where $\{\chi _{i}^{2}\}$ is a sequence of independent chi-squared random variables, each with one degree of freedom, and $\lambda _{1},\ldots ,\lambda _{m}$ are the eigenvalues of $({\mathbb {I}}_{m}-\varvec{Q_{s}})\varvec{M}$, where $\varvec{M}$ is a diagonal matrix of size *m* with diagonal elements $\{1, (m-1)/m, \ldots ,2/m,1/m\}$. $\square$

### **Theorem 3**

*Under the assumptions of * Theorem 1, $\mathfrak {D}_{m}(s)$*converges in distribution to*$\sum _{i=1}^{m}\lambda _{i}\chi _{i}^{2}$, *where*$\{\chi _{i}^{2}\}$*denotes a sequence of independent chi-squared random variables, each with one degree of freedom, and*$\lambda _{1},\ldots ,\lambda _{m}$*are the eigenvalues of*$\varvec{({\mathbb {I}}_{m}-Q_{s})M}$, *where*$Q_{s}$*is given in* Theorem 2*and*$\varvec{M}$*is a diagonal matrix of sizemwith diagonal elements*$\{m, m-1, \ldots ,1\}$.

### *Proof*

As in Mahdi and McLeod (2012), the determinant of the block partitioned matrix ${\mathcal {\hat{R}}}_{m}(s)$ is

15 $$|{\mathcal {\hat{R}}}_{m}(s)| = \prod _{\ell =1}^{m}(1-\hat{\eta}_{\ell} ^{2}(s)),$$

where $\hat{\eta}_{\ell} ^{2}(s)=\varvec{\hat{r}_{\ell s}^{\prime}}{\mathcal {\hat{R}}}_{(\ell -1)}^{-1}(s)\varvec{\hat{r}_{\ell s}}$ and $\varvec{\hat{r}_{\ell s}}=(r_{s},\ldots ,r_{\ell s})^{\prime}$. It follows that

16 $$-n\log |{\mathcal {\hat{R}}}_{m}(s)| = -n\sum _{\ell =1}^{m}\log (1-\hat{\eta}_{\ell} ^{2}(s)),$$

Taylor expansion of logarithmic function implies

17 $$\begin{aligned} \begin{aligned} -n\log |{\mathcal {\hat{R}}}_{m}(s)|&=n\sum _{\ell =1}^{m}\sum _{k=1}^{\infty}k^{-1}\hat{\eta}_{\ell} ^{2k}(s),\\&=n\sum _{\ell =1}^{m}(m-\ell +1)\hat{r}_{\ell s}^{2}+O_{p}(n^{-3}), \end{aligned} \end{aligned}$$

Following the same arguments in proof of Theorem 2, the asymptotic distribution of $-n\log |{\mathcal {\hat{R}}}_{m}(s)|$ is approximated by

18 $$-n\log |{\mathcal {\hat{R}}}_{m}(s)| \rightarrow \sum _{i=1}^{m}\lambda _{i}\chi _{i}^{2},$$

where $\{\chi _i^{2}\}$ is a sequence of independent chi-squared random variables, each with one degree of freedom, and $\lambda _{1},\ldots ,\lambda _{m}$ are the eigenvalues of $({\mathbb {I}}_{m}-\varvec{Q_{s}})\varvec{M}$, where $\varvec{M}$ is a diagonal matrix of size *m* with diagonal elements $m, m-1, \ldots ,1$. $\square$

It is worth noting that the $\mathfrak {D}_{m}(s)$ statistic may be seen as a weighted Ljung and Box (1978) considering of the residual autocorrelations at the seasonal lags $1s, 2s, \ldots ,ms$. It essentially has the same characteristics as $\tilde{Q}_{m}(s)$ with standardizing weights $3m(2m+1)^{-1},3(m-1)(2m+1)^{-1},\ldots ,3(2m+1)^{-1}$ using the seasonal residuals at lags $1s, 2s, \ldots ,ms$.

From the theorem on quadratic forms given by Box (1954, Theorem 3.1) it follows that $\tilde{Q}_{m}(s)$ and $\mathfrak {D}_{m}(s)$ can be approximated by gamma distribution or $a\chi _{b}^{2}$, where *a* and *b* are chosen to make the first two moments agree with those of exact distribution of $\tilde{Q}_{m}(s)$ and $\mathfrak {D}_{m}(s)$. Hence, $a=\sum \lambda _{i}^{2}/\sum \lambda _{i}$ and $b=(\sum \lambda _{i})^{2}/\sum \lambda _{i}^{2}$, where,

19 $$\begin{aligned} \begin{aligned} \sum _{i=1}^{m}\lambda _{i}&= \mathrm{tr\,}({\mathbb {I}}_{m}-\varvec{Q_{s}})\varvec{M},\\ \sum _{i=1}^{m}\lambda _{i}^{2}&=\mathrm{tr\,}({\mathbb {I}}_{m}-\varvec{Q_{s}})\varvec{M}({\mathbb {I}}_{m}-\varvec{Q_{s}})\varvec{M}. \end{aligned} \end{aligned}$$

where $\lambda _{1},\ldots ,\lambda _{m}$ are the eigenvalues of $({\mathbb {I}}_{m}-\varvec{Q_{s}})\varvec{M}$ and $\varvec{M}$ is a diagonal matrix of size *m* with diagonal elements $\{1, (m-1)/m, \ldots ,2/m,1/m\}$ for the statistic $\tilde{Q}_{m}(s)$ and diagonal elements $\{m, m-1, \ldots ,1\}$ for the statistic $\mathfrak {D}_{m}(s)$.

So that the seasonal portmanteau test statistic $\mathfrak {D}_{m}(s)$ may approximately distributed as $\chi _{b}^{2}$, where $b=3m(m+1)(4m+2)^{-1}-\nu$, whereas the seasonal test statistic $\tilde{Q}_{m}(s)$ can be approximated as Gamma with shape and scale

$$\begin{aligned} \alpha = \frac{(\sum w_{\ell} )^{2}}{2(\sum w_{\ell} ^{2}-\nu )}\,\quad \hbox {and}\,\quad \beta = \frac{2(\sum w_{\ell} ^{2}-\nu )}{\sum w_{\ell}}, \end{aligned}$$

respectively, where $\nu =p_{s}+q_{s}$ and $\{w_{\ell} \}$ is the sequence of weights satisfies $\sum _{\ell =1}^{\infty} w_{\ell} < \infty$.

## Simulation studies

The objective of our simulations is to explore the performance of the proposed portmanteau seasonal tests, $Q_{m}(s),\hat{Q}_{m}(s),\tilde{Q}_{m}(s)$, and $\mathfrak {D}_{m}(s)$, in finite samples and when the sample size grow. We study the empirical type I and type II error rates demonstrating the accuracy of the approximation distributions of the proposed seasonal tests in producing the correct sizes and conducting a power comparison studies. For each simulation experiment, we determine the critical values from the corresponding asymptotic distributions of the proposed seasonal test statistics. One can use the Monte-Carlo test procedures, as described by Lin and McLeod (2006) and Mahdi and McLeod (2012), to compute these critical values instead of using the approximation distributions. The simulations were run on a modern quad-core personal computer using the **R** package portes (Mahdi and McLeod 2015) and WeightedPortTest (Fisher and Gallagher 2012) that are available from the CRAN website (R Development Core Team 2015).

### Comparison of type I error rates

**Fig. 1:**
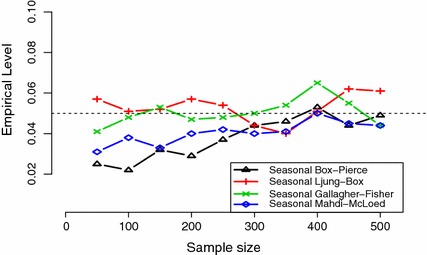
The empirical 5 % significance level of the seasonal portmanteau test statistics $Q_{m}(4),\hat{Q}_{m}(4),\tilde{Q}_{m}(4)$, and $\mathfrak {D}_{m}(4)$ at lag $m=10$, for a fitted SAR $(1)_{4}$ model to series with lengths $n=50,100,150,200,250,300,350,400,450$ and 500 simulated from a Gaussian quarter SAR model with a coefficient $\Phi = 0.3$

The empirical type I error rates at nominal levels 1, 5, and 10 % for the portmanteau seasonal test statistics using the approximation distributions based on $10^{4}$ simulations have been evaluated under the Gaussian SAR $(1)_{s}$ models where $s=4,12$. The results were summarized in Table 1 at lags $m=5$, and 15 and Fig. 1 at lag $m=10$. It is seen that seasonal portmanteau test statistic convergence to its asymptotic distribution increases as the sample size *n* increases from 50 to 500 and all proposed statistics have acceptable size levels compared to their nominal levels.

**Table 1:** The empirical 1, 5 and 10 % significance levels for different fitted SAR $(1)_{s}$ models, with different SAR coefficients $\Phi _{1}= 0.1,0.3, 0.5, 0.7,$ and 0.9, for the seasonal portmanteau test statistics $\hat{Q}_{m}(s),\tilde{Q}_{m}(s)$, and $\mathfrak {D}_{m}(s)$, where $s=4,12$, $n=200$ and lags $m=5,15$

	$\Phi _{1}$	$\hat{Q}_{5}(s)$	$\tilde{Q}_{5}(s)$	$\mathfrak {D}_{5}(s)$	$\hat{Q}_{15}(s)$	$\tilde{Q}_{15}(s)$	$\mathfrak {D}_{15}(s)$
$(s=4)\,\alpha =0.01$	0.1	0.010	0.013	0.007	0.013	0.012	0.008
	0.3	0.007	0.017	0.007	0.009	0.010	0.007
	0.5	0.009	0.010	0.008	0.014	0.007	0.009
	0.7	0.010	0.017	0.009	0.014	0.015	0.006
	0.9	0.016	0.009	0.009	0.019	0.005	0.007
$\alpha =0.05$	0.1	0.050	0.041	0.034	0.051	0.038	0.033
	0.3	0.043	0.051	0.039	0.035	0.040	0.037
	0.5	0.049	0.049	0.042	0.055	0.039	0.041
	0.7	0.049	0.070	0.041	0.068	0.050	0.041
	0.9	0.060	0.055	0.044	0.064	0.043	0.042
$\alpha =0.10$	0.1	0.091	0.081	0.077	0.109	0.070	0.070
	0.3	0.092	0.105	0.089	0.084	0.084	0.088
	0.5	0.095	0.087	0.082	0.101	0.081	0.081
	0.7	0.093	0.134	0.090	0.106	0.102	0.086
	0.9	0.122	0.107	0.085	0.113	0.090	0.088
$(s=12)\,\alpha =0.01$	0.1	0.018	0.018	0.006	0.014	0.011	0.005
	0.3	0.015	0.010	0.007	0.018	0.008	0.007
	0.5	0.015	0.018	0.010	0.016	0.012	0.008
	0.7	0.019	0.014	0.012	0.015	0.010	0.011
	0.9	0.023	0.015	0.008	0.022	0.013	0.009
$\alpha =0.05$	0.1	0.070	0.061	0.031	0.046	0.041	0.030
	0.3	0.068	0.063	0.044	0.067	0.041	0.040
	0.5	0.072	0.070	0.040	0.050	0.047	0.041
	0.7	0.075	0.076	0.039	0.069	0.054	0.037
	0.9	0.073	0.069	0.043	0.072	0.060	0.038
$\alpha =0.10$	0.1	0.119	0.126	0.083	0.088	0.091	0.080
	0.3	0.129	0.142	0.090	0.118	0.089	0.121
	0.5	0.133	0.140	0.102	0.091	0.104	0.099
	0.7	0.150	0.144	0.074	0.114	0.104	0.088
	0.9	0.141	0.140	0.088	0.130	0.120	0.111

### Power comparisons

Here, we conduct a power comparison simulation study between the proposed seasonal $\hat{Q}_{m}(s),\tilde{Q}_{m}(s),\mathfrak {D}_{m}(s)$ statistics where the critical values are calculated from the corresponding asymptotic distributions. Table 2 below provides the empirical power of these statistics when a series of length $n=200$ is generated from a 20 Gaussian SARMA $(2,2)\times (2,2)_{s}$ processes are inadequately fitted by SAR $(1)_{s}$ or SMA $(1)_{s}$, $s=4$ and 12, and tested at lag $m=10$. In each case, the test statistic with the largest power has been put in italic to assist the reader. The results in Table 2 indicate that the proposed tests are competitors to each others with no absolute known optimal test that is determined.

**Fig. 2:**
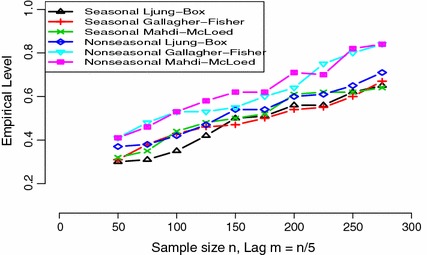
Empirical power as a function of sample size *n* and maximum lag $m= n/5$ comparing seasonal ($s=4$) to nonseasonal ($s=1$) tests, where series from nonseasonal ARMA (1,1) with $\phi _{1}=0.9$ and $\theta _{1}=0.8$ are generated, and a SMA $(1)_{4}$ model is fitted

To compare the empirical power of our proposed seasonal statistics with those classical statistics found in literature, we generated data from a nonseasonal ARMA (1,1) process $Z_{t}=0.9 Z_{t-1}+a_{t}-0.8 a_{t-1}$ and improperly fit a seasonal moving average SMA $(1)_{4}$. The results are presented on Fig. 2 where the power of these statistics is shown as a function of the sample size *n* and maximum lag $m=n/5$. We see that in this particular case, when the correlations at the nonseasonal lags are presented, the classical nonseasonal tests in most cases outperform the proposed nonseasonal statistics. For this reason, we recommend to restrict the use of our proposed seasonal test statistics as complementary (and not as an alternative) to other classical statistics found in literature.

**Table 2:** The empirical power for a nominal 5 % level test comparing the approximation distributions of the seasonal portmanteau test statistics $\hat{Q}_{m}(s),\tilde{Q}_{m}(s)$, and $\mathfrak {D}_{m}(s)$, at lag $m=10$ based on $10^{4}$ simulations. In each simulation, the SAR $(1)_{s}$ and SMA $(1)_{s}$ are fitted to data of series length $n=200$ generated from SARMA $(2,2)\times (2,2)_{s}$ models where asterisk (*) refers to NULL and $s=4,12$

Model	$\phi _{1}$	$\phi _{2}$	$\theta _{1}$	$\theta _{2}$	$\Phi _{1}$	$\Phi _{2}$	$\Theta _{1}$	$\Theta _{2}$	$\hat{Q}_{m}(4)$	$\tilde{Q}_{m}(4)$	$\mathfrak {D}_{m}(4)$	$\hat{Q}_{m}(12)$	$\tilde{Q}_{m}(12)$	$\mathfrak {D}_{m}(12)$
Fitted by SAR (1)														
1	*	*	*	*	*	*	−0.5	*	0.111	*0.133*	0.129	0.100	0.113	*0.119*
2	*	*	*	*	*	*	−0.6	0.3	0.333	0.301	*0.389*	0.301	0.301	*0.356*
3	*	*	*	*	0.7	*	−0.4	*	0.087	*0.091*	0.063	0.084	*0.090*	0.062
4	*	*	*	*	0.1	0.3	*	*	0.102	0.119	*0.120*	0.092	*0.095*	0.089
5	0.3	*	*	*	−0.35	*	*	*	*0.786*	0.663	0.652	*0.763*	0.641	0.661
6	0.4	*	*	*	*	*	−0.8	*	0.961	*0.998*	0.971	0.951	*0.996*	0.965
7	*	*	*	*	0.4	−0.6	0.3	*	0.371	0.401	*0.442*	0.363	0.396	*0.406*
8	0.7	0.2	*	*	−0.5	*	*	*	1.000	1.000	1.000	1.000	1.000	1.000
9	0.7	*	0.7	*	−0.8	*	*	*	*0.124*	0.096	0.075	*0.119*	0.094	0.066
10	0.1	0.3	*	*	*	*	−0.8	*	0.867	*0.918*	0.889	0.844	*0.907*	0.887
Fitted by SMA (1)														
11	*	*	*	*	0.5	*	*	*	0.126	0.148	*0.150*	0.111	*0.144*	0.143
12	*	*	*	*	*	*	−0.6	0.3	0.175	0.172	*0.264*	0.161	0.165	*0.244*
13	*	*	*	*	0.7	*	−0.4	*	0.382	*0.701*	0.665	0.378	*0.700*	0.662
14	*	*	*	*	0.1	0.3	*	*	0.113	0.100	*0.211*	0.109	0.098	*0.203*
15	0.3	*	*	*	−0.35	*	*	*	*0.760*	0.689	0.662	*0.755*	0.679	0.654
16	0.4	*	*	*	*	*	−0.8	*	0.958	*0.961*	0.913	0.953	*0.958*	0.908
17	*	*	*	*	0.4	*	0.3	*	0.204	*0.367*	0.265	0.201	*0.295*	0.257
18	0.7	0.2	*	*	−0.5	*	*	*	1.000	1.000	1.000	1.000	1.000	1.000
19	0.7	*	0.7	*	−0.8	*	*	*	0.632	0.761	*0.935*	0.628	0.757	*0.931*
20	0.1	0.3	*	*	*	*	−0.8	*	*0.779*	0.705	0.700	*0.770*	0.698	0.698

**Fig. 3:**
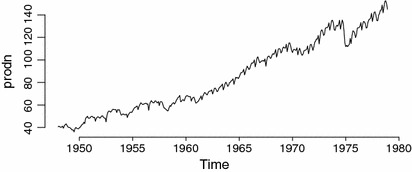
Monthly Federal Reserve Board Production Index data

## An empirical application

In this section, we make use of the monthly Federal Reserve Board Production Index data. Data is available from the R package astsa with the name prodn from January 1948 to December 1978 with 372 observations (Shumway and Stoffer 2011) and displayed in Fig. 3. All p-values from seasonal and nonseasonal tests suggest rejecting the null hypothesis, at the significance of 5 % level, that the seasonal and nonseasonal autocorrelations of the prodn series are equal to zero. Following Shumway and Stoffer (2011), we take the seasonal difference of the differenced production data $\nabla _{12}(Z_{t}-Z_{t-1})$ and apply the BIC criteria to select the preferred model SARMA $(2,0)\times (0,3)_{12}$. Here, we are not interested in selecting the best fitted model but the main objective of this application is to demonstrate that the proposed seasonal tests are useful for investigating whether the autocorrelations of the residual SARMA model at the seasonal period are different from zero.

**Fig. 4:**
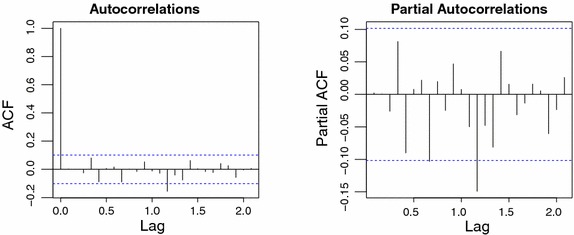
Diagnostic plots for residuals of fitted SARMA $(2,0)\times (0,3)_{12}$ to monthly difference of the differenced Federal Reserve Board Production Index data

A diagnostic check on the residual series is displayed in Fig. 4, and we note, as indicated by Shumway and Stoffer (2011), that there may be a small amount of nonseasonal autocorrelation still remained in the SARIMA $(2,1,0)\times (0,1,3)_{12}$ model (not at the multiple of the seasonal lags).

**Table 3:** The SARMA $(2,0)\times (0,3)_{12}$ model was fitted to the monthly difference of the differenced federal reserve board production index data

Test	$m=10$		$m=15$		$m=20$	
	$s=12$	$s=1$	$s=12$	$s=1$	$s=12$	$s=1$
$Q_{m}(s)$	0.822	0.114	0.381	0.030	0.574	0.069
$\hat{Q}_{m}(s)$	0.744	0.107	0.087	0.024	0.093	0.055
$\tilde{Q}_{m}(s)$	0.824	0.097	0.676	0.033	0.596	0.058
$\mathfrak {D}_{m}(s)$	0.623	0.057	0.520	0.076	0.570	0.054

The residuals of the fitted model are tested at the seasonal and nonseasonal lags using the portmanteau test statistics $Q_{m}(s),\hat{Q}_{m}(s),\tilde{Q}_{m}(s)$, and $\mathfrak {D}_{m}(s)$ approximations, where $s=1,12$ (for nonseasonal and seasonal respectively) and $m=10,15,$ and 20

We apply the approximation distribution tests for the p-values associated with $\alpha =5\,\%$ of $\hat{Q}_{m}(s),\tilde{Q}_{m}(s)$ and $\mathfrak {D}_{m}(s)$, on the residuals of the SARIMA $(2,1,0)\times (0,1,3)_{12}$ model, where $m=10,15$, and 20 are the lags at seasonal and nonseasonal periods $s=12,1$, respectively (Table 3). As seen in Table 3, all seasonal tests indicate that the SARIMA model is good in capturing the seasonal autocorrelations where no period autocorrelations are detected at seasonal lags 10, 15, and 20. On the other hand, as noted by Shumway and Stoffer (2011), we note that the classical nonseasonal tests (except that $\mathfrak {D}_{m}$) indicate that the model SARIMA $(2,1,0)\times (0,1,3)_{12}$ is inadequate where it does not capture the nonseasonal autocorrelations at lag $m=15$.

## Conclusion

Despite the popularity of the SARMA models in various economic and financial data, the goodness-of-fit portmanteau tests at multiple period lags $1s,2s,3s,\ldots ,ms$, where *m* is the largest lag considered for autocorrelation and *s* is the seasonal period, has not yet received as much attention as it should deserve. In literature, the classical nonseasonal portmanteau statistics Box and Pierce (1970), Ljung and Box (1978), Peña and Rodríguez (2002, 2006), Mahdi and McLeod (2012), Fisher and Gallagher (2012) and Gallagher and Fisher (2015) for testing the lack of fit of SARMA models would be misleading since they are only implementing at the nonseasonal lags $1,2,\ldots ,m$ ignoring the possibility of autocorrelations at seasonal lags of multiple period *s*. In this paper, we devise a new list of portmanteau statistics for seasonal time series using the asymptotic distribution of the residual autocorrelation at seasonal lags of multiple period *s*. We modify the classical nonseasonal portmanteau tests of the ARMA models mentioned above to the SARMA class with a case of $p,q\ll s$ and the roots of the equation $\phi ({B})\theta ({B})=0$ are not close to the unit circle. We provide simulation studies to demonstrate that the asymptotic tests are valid with satisfactorily performance in finite sample. In summary, in order to check the adequacy of time series models, we recommend to use the seasonal and nonseasonal versions of anyone of the portmanteau test statistics Box and Pierce (1970), Ljung and Box (1978), Peña and Rodríguez (2002, 2006), Mahdi and McLeod (2012), Fisher and Gallagher (2012) and Gallagher and Fisher (2015) as complementary to each other.
